# Pituitary Disease in *AIP* Mutation-Positive Familial Isolated Pituitary Adenoma (FIPA): A Kindred-Based Overview

**DOI:** 10.3390/jcm9062003

**Published:** 2020-06-26

**Authors:** Ismene Bilbao Garay, Adrian F. Daly, Nerea Egaña Zunzunegi, Albert Beckers

**Affiliations:** 1 Department of Endocrinology, Hospital Universitario Donostia, 20014 Donostia, Euskadi, Spain; ismene.bilbaogaray@osakidetza.eus (I.B.G.); nerea.eganazunzunegui@osakidetza.eus (N.E.Z.); 2 Department of Endocrinology, Centre Hospitalaire Universitaire de Liège, Liège Université, 4000 Liège, Belgium

**Keywords:** Familial isolated pituitary adenoma, AIP, GPR101, acromegaly, prolactinoma, pituitary apoplexy, genetics

## Abstract

Clinically-relevant pituitary adenomas occur in about 1:1000 of the general population, but only about 5% occur in a known genetic or familial setting. Familial isolated pituitary adenomas (FIPA) are one of the most important inherited settings for pituitary adenomas and the most frequent genetic cause is a germline mutation in the *aryl hydrocarbon receptor-interacting protein* (*AIP*) gene. *AIP* mutations lead to young-onset macroadenomas that are difficult to treat. Most are growth hormone secreting tumors, but all other secretory types can exist and the clinical profile of affected patients is variable. We present an overview of the current understanding of *AIP* mutation-related pituitary disease and illustrate various key clinical factors using examples from one of the largest *AIP* mutation-positive FIPA families identified to date, in which six mutation-affected members with pituitary disease have been diagnosed. We highlight various clinically significant features of FIPA and *AIP* mutations, including issues related to patients with acromegaly, prolactinoma, apoplexy and non-functioning pituitary adenomas. The challenges faced by these *AIP* mutation-positive patients due to their disease and the long-term outcomes in older patients are discussed. Similarly, the pitfalls encountered due to incomplete penetrance of pituitary adenomas in *AIP-*mutated kindreds are discussed.

## 1. Introduction

Clinically relevant pituitary adenomas have a prevalence of about one per thousand in the general population and are an important cause of morbidity due to the combined impact of hormonal dysfunction and tumoral mass effects [1,2]. About 5% of cases occur in an inherited or familial setting; some of these form a component of multi-site tumor syndromes like multiple endocrine neoplasia (MEN) types 1 and 4 and Carney complex, among others [3]. Alternatively, pituitary adenomas can occur alone in a family setting, as part of familial isolated pituitary adenomas (FIPA) [4,5,6]. Up to 20% of FIPA kindreds have germline mutations of the aryl hydrocarbon receptor-interacting protein (*AIP*) gene [7,8]; rare cases of FIPA can also be caused by Xq26.3 microduplications that include the gene GPR101 [9,10]. *AIP* mutations lead to pituitary adenomas that have a distinctive clinical profile with a significantly younger onset, more aggressive tumor growth and larger tumor diameter at diagnosis, as compared with non-*AIP*-associated pituitary tumors [11]. As somatotropinomas secreting excess growth hormone (GH) are the most frequently encountered pituitary adenoma in the setting of *AIP* mutations, the young onset and aggressive tumor growth mean that *AIP* mutations are the main genetic cause of pituitary gigantism (about 30% of cases) [12]. Pituitary adenomas caused by *AIP* mutations are of great clinical relevance as they are difficult to treat due to large tumor size and high hormonal secretion levels; additionally, *AIP* mutations confer relative resistance to first-generation somatostatin analogs [11].

Results from large international studies have identified many hundreds of FIPA kindreds and have characterized a wide variety of *AIP* mutations and deletions affecting various families and individuals [13,14]. Within these families, the clinical presentations can vary beyond the more typical picture of the young-onset acromegaly patient to include other pituitary tumor types. Also, clinical and genetic screening of families can reveal occult pituitary tumors or individuals with long-term sequalae of early life pituitary disease. These issues represent a challenge to the clinician following such patients as the approach to each patient and mutation carrier must be individualized. To illustrate the various ways that *AIP* mutations can present in the FIPA setting, we describe and discuss the experiences of a large kindred with six affected individuals with *AIP* mutations and pituitary adenoma-related disease.

## 2. Experimental Section

### 2.1. Index Case

**Figure 1:**
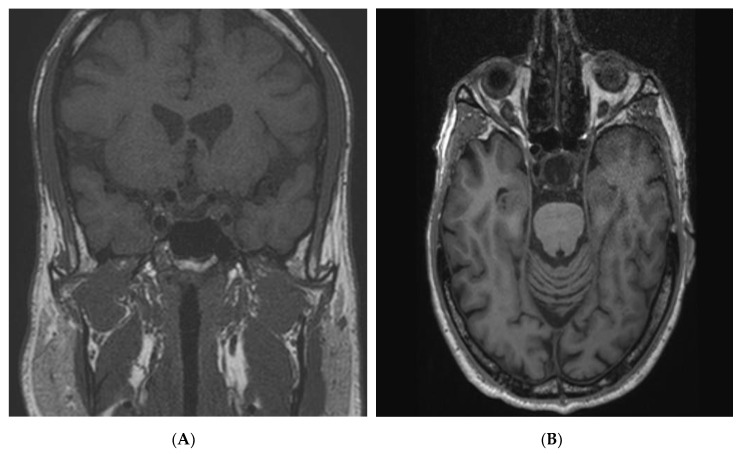

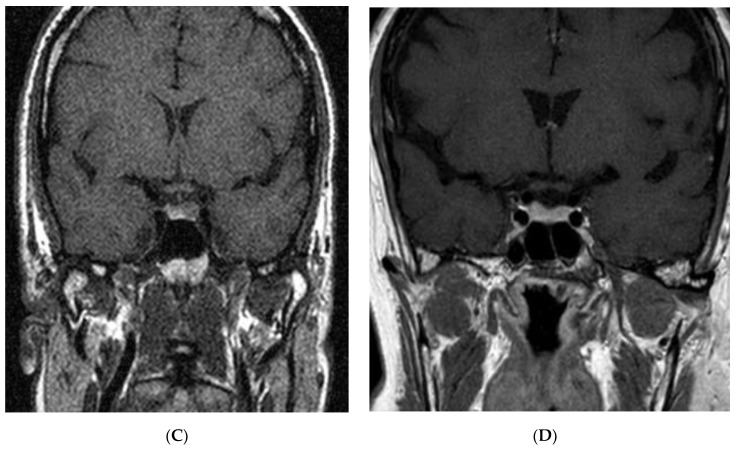
Magnetic resonance imaging (MRI) results in *AIP* mutation-positive members of the familial isolated pituitary adenoma (FIPA) kindred. Panel (**A**) shows the last follow-up coronal T1 weighted MRI decades after neurosurgery and external beam radiotherapy for the treatment of acromegaly. The image reveals a linear residue of pituitary tissue on the sellar floor and retraction of the optic chiasma. Panel (**B**) is a coronal T1 weighted MRI that demonstrates a cystic lesion measuring 19 × 14 × 7 mm in individual III-1 >50 years after surgery and radiotherapy for young onset acromegaly. Panel (**C**) is a coronal T1 weighted MRI that illustrates a recurrent pituitary adenoma measuring 10 × 9 × 8 mm adenoma in individual III-7, who was diagnosed with a prolactinoma 35 years previously. The recurrent tumor was associated with prolactin hypersecretion, mild insulin-like growth factor-1 (IGF-1) elevation and acromegalic features, leading to a diagnosis of a mixed tumor that responded to cabergoline. Panel (**D**) shows a coronal T1 weighted image of the normal pituitary in individual III-4, an *aryl hydrocarbon receptor interacting protein* (*AIP*) gene mutation carrier who is the mother of the index patient.

A 39-year-old man attended the endocrinology outpatient department for routine follow-up. He had chronic hypopituitarism involving the gonadal, thyroid and adrenal axes, for which he was receiving testosterone gel 50 mg/day, levothyroxine 200 μg/day and hydrocortisone 20 mg/day. He had developed hypopituitarism secondary to the treatment of a GH secreting macroadenoma diagnosed 23 years previously at the age of 16. He had been diagnosed at that time with acromegaly following a one-year history of frontal headaches and worsening visual disturbances accompanied by acral overgrowth and increasing height (183 cm). A CT scan at the time identified a pituitary macroadenoma with suprasellar extension. He was operated via a transcranial approach and, as he had increased GH and IGF-1 levels postoperatively, he was treated with external beam radiotherapy. His postoperative MRI showed a 10 mm residue. Over time, his GH and IGF-1 levels gradually came under control and at the last outpatient visit, his nadir GH after an oral glucose load was <0.1 ng/mL and his IGF-1 was 137.1 ng/mL (normal range for age and sex: 135–353 ng/mL). In keeping with the treatment and hormonal profile, the current MRI showed no evidence of pituitary adenoma and a small linear residue of pituitary tissue on the sellar floor, accompanied by retraction of the optic chiasma (Figure 1A). 

*Commentary:* The clinical history of the index patient typifies many aspects of *AIP* mutation-related pituitary adenomas. Patients with *AIP* mutations are at increased risk of developing early onset pituitary macroadenomas; the median age at diagnosis is 21 years and nearly 80% are diagnosed before the age of 30 [13]. A majority of affected patients with *AIP* mutated pituitary adenomas are male [11]. *AIP* mutation-related pituitary adenomas are usually somatotropinomas, as in this patient’s case, or mixed GH and prolactin secreting tumors [13]. These begin during childhood or adolescence in 52.2% of individuals, as compared with only 4.3% of acromegaly patients without *AIP* mutations [13]. The patient did not receive medical treatment, but rather, following an unsuccessful transcranial operation many years ago, was referred directly for radiotherapy. When hypopituitarism is present, patients with *AIP* mutation-related somatotropinomas have a significantly higher number of deficient axes compared with non-*AIP* mutated acromegaly patients [11].

### 2.2. Family Investigations: AIP in FIPA

The patient had a strong family history of pituitary disease. One uncle and one aunt were known to be affected with pituitary adenomas and the family had been investigated and were negative genetically for multiple endocrine neoplasia type 1 (MEN1). Therefore, the kindred were diagnosed with FIPA. 

**Figure 2:**
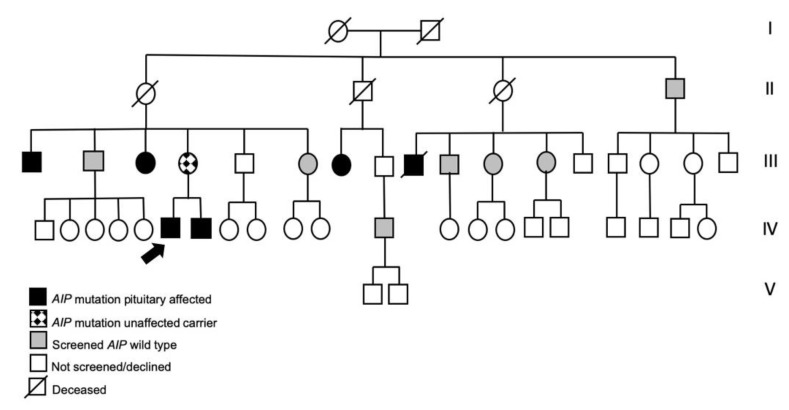
Genealogic tree of the *aryl hydrocarbon receptor interacting protein* (*AIP*) gene mutation-positive familial isolated pituitary adenoma (FIPA) kindred. The index case (IV-6) is indicated by a black arrow. Generations I through V are highlighted on the right of the figure.

Investigations in the index case and the family were undertaken. The data collection was approved by the Ethics Committee of the CHU de Liège (Clinical Trial Registration Numbers: B7072006587, B70720109577). A family tree was constructed, as shown in Figure 2 and the index case is indicated as individual IV-6. Historical medical records were assessed to extract relevant clinical features at presentation. Comprehensive clinical details collected included signs and symptoms, vital signs and physical examination; particular emphasis was placed on clinical indicators of pituitary dysfunction (headache, visual disturbance, physical features of hormonal hypersecretion or deficiency). Hormonal studies included comprehensive pituitary hormonal assays focused on GH, prolactin, luteinizing hormone (LH), follicle-stimulating hormone (FSH), adrenocorticotropic hormone (ACTH), thyroid stimulating hormone (TSH) and their related axes (insulin-like growth factor-1 (IGF-1), cortisol, sex hormones and T3/T4). Neuroradiological imaging was performed using magnetic resonance imaging (1.5 or 3 Tesla). Following informed consent, genetic studies were offered in the kindred, focusing on the *AIP* gene; sequencing was performed on peripheral blood leukocyte DNA as described previously [7,8].

Genetic investigation of the *AIP* gene in the index case revealed a heterozygotic c.543delT mutation. In the kindred, a total of 14 individuals agreed to testing for the c.543delT *AIP* mutation. Screening was performed, where possible, in a cascade manner from older to younger generations in order to focus testing on members with potential mutation carrier status. As noted above, there were two previously-identified members of the FIPA kindred with a diagnosis of pituitary adenomas.

Overall, genetic investigations revealed a total of seven *AIP* mutation-positive individuals (six with pituitary adenomas or a strong history of apoplexy) among 14 members of the kindred that were tested (Figure 2).

*Commentary: AIP* mutations usually present in the setting of FIPA (68.2%) and most are GH secreting or mixed GH/prolactin secreting tumors [13]. Genetic testing is recommended in relatives of known *AIP* mutation carriers, as part of a comprehensive clinical assessment that focusses on signs and symptoms suggestive of a pituitary adenoma or hormonal dysregulation [3]. Given the discovery of an *AIP* mutation in FIPA, kindred, clinical, hormonal and genetic assessments were undertaken throughout the family. The familial *AIP* mutation was confirmed in the two previously known pituitary adenoma family members and new affected cases and mutation carriers were also identified on screening:

### 2.3. Acromegaly

Individual III-1 is the index patient’s 80-year-old maternal uncle, who was heterozygous for the c.543delT *AIP* mutation. He was diagnosed with a pituitary tumor at the age of 16, which had led to increased growth. His height was 170 cm, but this was complicated by severe scoliosis that occurred in adolescence. He underwent cranial surgery and radiotherapy >50 years previously overseas and detailed pathological records regarding tumor characteristics are unavailable. He had not attended for follow-up for many years and on his most recent hormonal profile he was found to have central hypothyroidism, which was corrected. His nadir GH was 0.3 ng/mL and his IGF-1 was low at 76.2 ng/mL (normal range: 135–353 ng/mL); his other hormonal axes were normal. MRI revealed a cystic sellar lesion measuring 19 × 14 × 7 mm (Figure 1B), which was accompanied by a frontal parasagittal meningioma. His medical history was positive for a bronchial carcinoid with atypical features on pathology (necrosis, 2 mitoses/hpf; immunohistochemistry Ki-67: 4–5%; TTF1 negative, CK AE1/AE3 positive; synaptophysin positive); tissue was not available for *AIP* studies.

*Commentary:* Much of the published information about *AIP* mutation-related pituitary adenomas relates to the initial clinical characteristics of patients and short- to medium-term follow-up. Patients with *AIP* mutation-related acromegaly are significantly more difficult to treat than wild-type controls, with a cumulatively greater number of treatment modalities required [11]. Multiple surgeries and radiotherapy can lead to hypopituitarism and *AIP* mutation-associated acromegaly patients have significantly more pituitary axes affected by hypopituitarism than non-*AIP* mutated acromegaly controls [11]. Although individual III-1 had an early-onset case of acromegaly treated more than 50 years previously, his outcome at the last follow-up was relatively good, with a mild GH deficiency and readily-correctable hypothyroidism. This underlines the fact that while *AIP* mutations confer a relatively treatment-resistant form of acromegaly, good outcomes and disease control can be achieved in a proportion of patients. The concomitant finding of a bronchial carcinoid and a meningioma in individual III-1 is interesting and the issue of extra-pituitary tumors is discussed below in Section 2.6.

### 2.4. Prolactinoma and mixed tumors

Individual III-7: On family questioning it was discovered that the first cousin, once-removed, of the index patient had a history of having a pituitary adenoma. This 65-year-old woman had been diagnosed with an 8 mm prolactinoma at the age of 30 following investigation for secondary amenorrhea. At presentation, her prolactin levels were elevated at up to 253 ng/dL and following treatment with bromocriptine (7.5 mg/day), prolactin was normalized, her tumor shrank and her menses returned. After extended treatment and normal prolactin levels, bromocriptine was withdrawn at the age of 45 and she was later lost to follow-up.

During investigation of the family following the *AIP* mutation diagnosis in the index patient, she was re-contacted and when the prolactinoma history was learned of, she was offered hormonal and imaging screening in addition to genetic testing. In the intervening years she had been diagnosed with type II diabetes mellitus and hypertension. On examination, she had acromegalic features and complained of increased perspiration. Hormonal studies revealed hyperprolactinemia and an IGF-1 level of 373 ng/mL (normal range: 72–261 ng/mL). Her GH was suppressed to <1 ng/mL on an oral glucose tolerance test, but was not below the limit of detection. A pituitary MRI revealed an intrasellar pituitary adenoma of 10 × 9 × 8 mm in size (Figure 1C). The familial c.543delT *AIP* mutation was discovered on genetic testing. She was commenced on cabergoline, reaching 2 mg weekly and her prolactin decreased from 156.8 to 16.3 ng/mL (normal range: 4.0–15.2 ng/mL). Interestingly, her elevated IGF-1 also normalized with cabergoline treatment (falling from 373 to 256 ng/mL). After two years’ follow-up on cabergoline 2 mg/week, her prolactin and IGF-1 remained normal and her pituitary tumor shrank to 5 × 6 × 4.8 mm in size.

*Commentary:* Prolactinomas are a known, if less frequent (14.5%), manifestation of *AIP* mutation-related pituitary disease [13]. While few data have been published, they are more likely to be large and aggressive prolactinomas, with a relatively poor response to dopamine agonists [11,14,15]. Also, *AIP-*mutation-related prolactinomas—like all resistant prolactinoma types—more frequently affect males, as opposed to sporadic prolactinomas that are seen overwhelmingly in females. Individual III-7 was diagnosed at the age of 30 after some years of secondary amenorrhea and she responded well to bromocriptine. As such, her characteristics initially were those of a typical non-*AIP* mutation mutation-related prolactinoma in a female of childbearing age and her therapeutic response to a habitually-used dose of a dopamine agonist was excellent. After withdrawing bromocriptine following 15 years of therapy, she suffered a late recurrence of the pituitary adenoma, which, interestingly, was associated with both prolactin hypersecretion and elevated IGF-1. This suggests that she had a mixed GH- and prolactin-positive tumor at the time of recurrence, which is more typical of *AIP* mutation-related pituitary adenomas. Interestingly her prolactin and IGF-1 both responded to cabergoline at the upper end of the labeled dose range. The tumor, however, shrank by about 50% in size, unlike her original prolactinoma, which shrank completely on bromocriptine. Due to the lack of GH and IGF-1 results from the time of her original diagnosis, we do not know if there was always evidence of mixed hormone secretion or if this case represents an evolution from prolactinoma to mixed GH/prolactin-secreting adenoma.

### 2.5. Non-Functioning Pituitary Adenomas

Individual III-3 is the 74-year-old maternal aunt of the index case who was also heterozygous for the c.543delT *AIP* mutation. She presented initially to a gynecologist at the age of 28 with a two-year history of amenorrhea. She was treated with estrogen therapy for 10 years but no other investigations were conducted at the time. On stopping treatment her amenorrhea returned but she did not seek further medical advice. At the age of 48 she complained of headaches and visual disturbances and a CT scan of the brain was performed at another institution; she was diagnosed at the time with a non-functioning pituitary macroadenoma. She underwent a transcranial resection that was accompanied by external beam pituitary irradiation. At the time of the current genetic analysis, she had not attended an endocrine follow-up for more than 20 years. On physical examination, she had no features of acromegaly. An MRI showed a pituitary tumor residue of <10 mm in diameter. Laboratory studies showed hypopituitarism affecting her thyroid axis, while her gonadotropins were low. She was started on levothyroxine and is currently well, having regular follow-up. 

*Commentary:* Non-functioning pituitary adenomas comprise a minority of *AIP* mutation mutation-related pituitary adenomas, they constituted 6.5% of cases in our comprehensive review [13]. They can present in FIPA families or as rare sporadic cases. In the FIPA setting, they are usually seen in heterogeneous kindreds with GH or prolactin secreting tumors, but *AIP* mutation-positive FIPA families with homogenous presentation of non-functioning adenomas have been reported [14,16]. In the sporadic setting, non-functioning pituitary adenomas associated with *AIP* mutations share the same early onset of disease and large tumor size seen in *AIP-*associated acromegaly and prolactinoma [11,15,17]. In individual III-3, the symptom onset (amenorrhea) occurred in her mid-20s, but a diagnosis of a pituitary macroadenoma was not made until more than two decades later. Her clinical course mirrors that of other reported cases with *AIP* mutations in that while she was not cured by initial surgery, following radiotherapy, and during long-term follow-up, the tumor size was controlled without regrowth [11].

### 2.6. Pituitary Apoplexy and Other Tumors in AIP Mutation-Positive Patients

Individual III-9: As part of the family genetic study, this 62-year-old man was contacted and offered clinical assessment and *AIP* testing. On examination he was noted to be tall (197 cm) and he had a hypogonadal phenotype. He denied any past medical history but noted that he had been much taller than his peers during late childhood to mid-adolescence. He recalled that at the age of 16, his pubertal development stopped and he gradually lost his male pattern pubic hair distribution. Hormonal testing revealed hypopituitarism involving the somatotrope, thyroid and gonadal axes. Pituitary MRI showed an empty sella with erosion of the sellar floor. *AIP* testing revealed the familial c.543delT mutation. He received replacement therapy for his thyroid and gonadal insufficiencies (he declined GH replacement). Upon follow-up, at the age of 67, he was diagnosed with an adenocarcinoma of the transverse colon (pT3N2M1). This responded poorly to surgery and chemotherapy and he died due to metastatic progression.

*Commentary:* Patients with *AIP* mutation mutation-related pituitary adenomas have early disease onset and usually present with a macroadenoma. This implies that once tumorigenesis begins, tumor growth is rapid, as has been captured during serial MRI recently [18]. Another strong indication that *AIP* mutation-related pituitary adenomas grow rapidly comes from the reports of pituitary apoplexy seen in this population [11,14,16,19]. Indeed, *AIP* mutations can present with familial pituitary apoplexy as the only clinical disease manifestation [20]. Individual III-9 had a clinical history that is highly suggestive of pituitary apoplexy occurring against the backdrop of gigantism due to a somatotropinoma in late childhood/adolescence. Although he did not recall details suggestive of acute apoplexy >45 years before (headache, nausea, visual or oculomotor disturbances), this might have been due to a subclinical presentation, which has been reported [21,22,23]. He had tall stature and evidence of sellar erosion on MRI, which was associated with multi-axis pituitary insufficiency and noted pubertal arrest from the mid-teens. Post-apoplexy hypogonadism would also contribute to enhanced growth due to late fusion of the epiphyseal growth plates.

Since it was first identified in 2006 as a pituitary adenoma risk gene by Aaltonen’s group in Helsinki, much interest has been focused on whether *AIP* might be associated with tumor risk in other endocrine and non-endocrine organs. Unlike “classical” multiple endocrine neoplasia (MEN) risk genes such as *MEN1*, *PRKAR1A* or *CDKN1B*, which lead to multi-site tumorigenesis syndromes, *AIP* mutations only manifest to a reliable degree in the pituitary. Screening studies in other sporadic cancers like breast and colon or in non-*MEN1-*related MEN patients have shown no definitive link to *AIP* variants [24,25,26]. However, in the course of clinical investigations of FIPA families, many individuals with non-pituitary tumor associations (e.g., thyroid cancer, meningioma, and adrenal cortical carcinoma) within the kindred have been identified, including some with loss of heterozygosity at the *AIP* locus in tumoral tissue [13,18,27,28,29]. In the current kindred, various tumors in *AIP* mutation-affected patients were seen, including metastatic colorectal cancer, bronchial carcinoid and a meningioma. In the case of the colorectal cancer in individual III-9, which occurred after the diagnosis of the *AIP* mutation, it was possible to study this cancer for *AIP* status. Study of the resected colorectal tumor specimen demonstrated low levels of *AIP* immunohistochemical staining and there was no loss of heterozygosity at the *AIP* local in colorectal tumor DNA. These data indicate strongly that the colorectal cancer was not associated with the germline *AIP* mutation; moreover, while *AIP* immunostaining is a valuable marker in colorectal cancer, increased *AIP* staining intensity is associated with poorer outcome [30].

### 2.7. Screening and Investigation of AIP Mutation Carriers

A further two individuals were found to be positive for the familial *AIP* mutation. The first (individual III-4) was the 71-year-old mother of the index case. Clinical, biochemical and radiological screening were within normal limits initially and during long-term follow-up (Figure 1D). The second *AIP* mutation carrier (individual IV-7) was the 37-year-old brother of the index case. He was entirely asymptomatic and has normal clinical findings and hormonal testing. On MRI, a 6 mm diameter pituitary adenoma was identified. He remains asymptomatic on follow-up, without any change in size of his pituitary adenoma or hormonal abnormalities. Cascade screening studies were practically useful as they permitted the identification of one individual in generation II, five in generation III and one in generation IV who were wild-type for *AIP*. In total, a further 23 offspring of these unaffected non-carriers could be excluded from *AIP* mutation-related follow-up investigations, which has a number of advantages in terms of avoided stresses to individuals and unnecessary costs relating to hormonal and radiological testing.

*Commentary:* When an individual with an *AIP* mutation undergoes clinical and hormonal studies that are normal but nevertheless MRI reveals a pituitary adenoma, it raises a series of questions regarding etiology and follow-up. This is the case we encountered in the *AIP* mutation-positive individual IV-7, the brother of the index patient. His investigations demonstrated a pituitary microadenoma that was clinically and hormonally silent and did not grow over the course of some years of follow-up. The temptation is to classify the pituitary adenoma in this *AIP* mutation affected person as being *AIP*-related, however, several issues argue strongly against it. It must be recalled that pituitary adenomas occur at a high frequency in the general population, with one in five people having an unsuspected adenoma either at autopsy or on MRI performed for another reason (i.e., head injury). The overwhelming majority of these so-called incidentalomas are non-functioning adenomas of a few millimeters in diameter, which very rarely progress. MRI based screening studies of asymptomatic carriers of *AIP* or *MEN1* mutations unsurprisingly encounter multiple individuals with such small adenomas that do not grow or secrete hormones. In *AIP* mutation carriers, as seen in individual IV-7, small non-functioning pituitary tumors in an adult are not the typical presentation, which is usually young and with large GH- or GH/prolactin-secreting adenomas. Clinical context is important. Hence, in MRI screening of *AIP* mutation carriers, the finding of a microadenoma in a child/adolescent/young adult or an adenoma with hormonal abnormalities or rapid growth argues for an *AIP-*related etiology. As typical aggressive *AIP* mutation-related pituitary adenomas are only exceptionally (if ever) diagnosed after the age of 30, small, non-progressing, non-functioning adenomas in adult *AIP* mutation carriers are potentially more likely to be incidentalomas. The molecular features of early pituitary adenoma formation in the setting of *AIP* mutations are not fully understood; hence it is possible that some smaller adenomas could form in mutation carriers but subsequently fail to develop aggressive characteristics, thereby contributing to the incomplete penetrance seen in this condition.

## 3. Discussion

FIPA is one of the most frequent familial or inherited forms of pituitary adenomas and currently only two genetic causes have been identified: *AIP* germline mutations in about 20% of cases, and chromosome Xq26.3 duplications in exceptionally rare cases of X-LAG [31]. *AIP* mutations are associated with pituitary adenomas that usually present at a significantly younger age and with larger tumors than in non-*AIP-*associated adenomas [11]. They usually are somatotropinomas or mixed GH and prolactin secreting adenomas and this secretory profile, combined with the young age at onset (childhood and adolescence) means that *AIP* mutations are the most frequent known cause of pituitary gigantism [12]. These characterizations of typical FIPA and *AIP* related-pathology come from large international series of hundreds of patients [11,13,14]; in the clinical setting, more variability is seen, as in the FIPA kindred reported here. Clinicians need to be aware of the possibility of less typical pituitary presentations, such as prolactinoma or potential cases of pituitary apoplexy. Indeed, such conditions can occur at an early age but may not be linked to the underlying genetic condition until decades later. Also, the penetrance of clinical disease in *AIP-*mutated kindreds is incomplete and construction of a detailed family tree beyond the first-degree relatives of an index case is crucial to capture all possible cases (including apoplexy or hypopituitarism of unknown origin). The importance of mixed GH and prolactin secreting adenomas in *AIP-*mutated patients is also highlighted by the experience of one case in the kindred whose profile suggests that mild IGF-1 excess might have developed over time. Such instances of mild mixed IGF-1 and prolactin excess that are successfully treated with dopamine agonists have been noted previously in *AIP-*associated FIPA families [32]. During planned genetic investigations, it must be borne in mind that not all pituitary adenomas found in *AIP-*mutated FIPA kindreds are necessarily definitively linked to the genetic mutation. Small incidentally found non-secreting pituitary adenomas occur in up to one in five of the general population [33]. Therefore, in *AIP* mutation carriers undergoing MRI screening, caution should be exercised before definitively labeling such tumors as being “*AIP* related” pituitary adenomas, as such cases may behave in a manner that is indistinguishable from other incidentalomas on follow-up. In support of this, recent data from screening analyses suggest that nearly half of prospectively diagnosed pituitary adenomas in *AIP* mutation carriers are non-functioning microadenomas; these non-functioning tumors are usually not associated with hormonal abnormalities and rarely receive any treatment during long-term follow-up [34]. Screening, therefore, should be focused particularly on identifying undiagnosed tumors in young *AIP* mutation carriers from childhood to early adulthood. Finally, the clinical phenotype seen in FIPA related to *AIP* appears to be largely isolated to pituitary adenomas and their consequences. Despite a number of associated intracranial and other tumors being reported in the literature, they rarely have associated tumoral genetic studies supportive of an *AIP-*related role, as was confirmed in a case of metastatic colorectal carcinoma in the current FIPA family. Detailed studies of individual large kindreds like this one can be illustrative of the issues and choices faced by endocrinologists, geneticists and the family members themselves when an *AIP* mutation and pituitary adenomas are discovered.
